# Bayesian Spatial Joint Model for Disease Mapping of Zero-Inflated Data with R-INLA: A Simulation Study and an Application to Male Breast Cancer in Iran

**DOI:** 10.3390/ijerph16224460

**Published:** 2019-11-13

**Authors:** Naeimehossadat Asmarian, Seyyed Mohammad Taghi Ayatollahi, Zahra Sharafi, Najaf Zare

**Affiliations:** 1 Department of Biostatistics, School of Medicine, Shiraz University of Medical Sciences, Shiraz 7134845794, Iran; ns.asmarian@gmail.com (N.A.); zahra10926@gmail.com (Z.S.); najafzare@sums.ac.ir (N.Z.); 2 Infertility Research Center, Shiraz University of Medical Sciences, Shiraz 7134814336, Iran

**Keywords:** BYM2 model, disease mapping, INLA, joint model, male breast cancer, penalized complexity prior, zero-inflated Poisson model

## Abstract

Hierarchical Bayesian log-linear models for Poisson-distributed response data, especially Besag, York and Mollié (BYM) model, are widely used for disease mapping. In some cases, due to the high proportion of zero, Bayesian zero-inflated Poisson models are applied for disease mapping. This study proposes a Bayesian spatial joint model of Bernoulli distribution and Poisson distribution to map disease count data with excessive zeros. Here, the spatial random effect is simultaneously considered into both logistic and log-linear models in a Bayesian hierarchical framework. In addition, we focus on the BYM2 model, a re-parameterization of the common BYM model, with penalized complexity priors for the latent level modeling in the joint model and zero-inflated Poisson models with different type of zeros. To avoid model fitting and convergence issues, Bayesian inferences are implemented using the integrated nested Laplace approximation (INLA) method. The models are compared according to the deviance information criterion and the logarithmic scoring. A simulation study with different proportions of zero exhibits INLA ability in running the models and also shows slight differences between the popular BYM and BYM2 models in terms of model choice criteria. In an application, we apply the fitting models on male breast cancer data in Iran at county level in 2014.

## 1. Introduction

In recent years, spatial statistical methods and spatial models for smoothing disease rates are widely applied within epidemiology and ecology studies. Bayesian hierarchical models for the class of latent Gaussian models (LGMs) are frequently used in disease mapping. In fact, Bayesian hierarchical models improve the estimates of log risk by neighboring regions information in the spatial structured component as well as region variation in the unstructured component [1]. Besag, York and Mollié (BYM) model using Markov chain Monte Carlo (MCMC) method is a Bayesian hierarchical model that is widely used in disease mapping (see for example Ebrahimipour et al. [2] and Sharafi et al. [3]).

Unfortunately, the MCMC simulation methods for LGMs, in computing complex posterior distributions with large datasets, are time-consuming and problematic in convergence [1,4,5]. As to improving MCMC algorithms, several MCMC sampling strategies have been suggested; for example, methods based on the Metropolis-adjusted Langevin algorithm (MALA), methods based on Hamiltonian mechanics (HMC) and the single-block strategy [6]. However, despite all these developments, MCMC sampling techniques for LGMs suffer from slow convergence and poor mixing for large and complex models [4,5]. In order to solve these issues, Rue et al. [5] proposed an algorithm for full Bayesian inference, so-called integrated nested Laplace approximation (INLA). Carroll et al. [7] compared INLA and MCMC methods for hierarchical Poisson modeling in disease mapping via R and OpenBUGS. With the same prior distributions, they found the equivalent results using both methods, and by simulation they proved that INLA is computationally faster and more precise than MCMC. Simpson et al. [8], by using INLA, proposed a re-parameterization of the BYM model (BYM2), so as to make priors interpretable and transferable, using penalized complexity (PC) priors and scaled spatial component. Blangiardo and Cameletti [1] showed the ability of INLA in running complex models, explained spatial and spatiotemporal Bayesian models in several fields and implemented code of numerous practical examples with spatial structure in R package R-INLA.

Spatial models for count data are often based on Poisson distribution. However, Poisson distribution might be inappropriate when the number of zeros in data is more than expected (zero-inflated data). To overcome this, zero-inflated Poisson (ZIP) distribution can be used for modeling this type of data. ZIP distribution is a proportion of zeros in combination with Poisson distribution [9]. On the other hand, models which are based on ZIP distribution can be combined with any spatial Bayesian hierarchical model for count data. Hence, Agarwal et al. [10] employed the ZIP model with a spatial Bayesian hierarchical model and showed the model fitting on Isopod nest burrows data in a Bayesian framework. Then, Gschlößl and Czado [11] overviewed spatial regression models with Poisson, generalized Poisson and negative Binomial distributions for zero-inflated count data in a hierarchical Bayesian point of view. They also showed the models on the number of invasive meningococcal disease cases in Germany in 2004. Arab [12] reviewed hurdle and ZIP models with spatial and spatiotemporal structures and used the models to analyze five-year counts of the confirmed cases of Lyme disease in Illinois at the county level with the INLA method.

In most published studies regarding spatial modeling zero-inflated count data of disease and mortality, so as to reduce complexity, spatial component was considered just for the occurrence or for the number of occurrences in a single model [10,13]. On the other hand, when responses come from two different distributions (disease number and occurrence) and we have two models for each response that are affected by common factor (spatial), it is better to use joint model. Joint models, as a way to fit models better, to control all uncertainty sources and to provide accurate inference, have been expanded over the past decade (see for example Blangiardo and Cameletti [1], Zhu et al. [14] and Illian et al. [15]). In the same vein, we expand the class of joint models using the shared spatial random effect for mapping zero-inflated count data. In a spatial joint model structure, random spatial effects can be formulated simultaneously for both the occurrence and the number of occurrences. Incorporating spatial structure in the joint model reduces variability, since information may be borrowed across different datasets, but increases the complexity of a model [15]. Such models might become unstable and face convergence issues by MCMC methods. However, the complex posterior distributions of spatial joint models can be defined and run by the INLA approach [1,4,15]. 

In our study, it is assumed that zero-inflated data come from two distributions: Bernoulli and Poisson (one for the occurrence and one for the number of occurrences). In this study, we account for spatial random effect in a joint model consisting of logistic and log-linear model to model both parameters of distributions simultaneously. In order to solve the mentioned issues, the parameters of the models are estimated by the INLA method via R-INLA. As spatial modeling, the BYM and BYM2 models, which have different ways of assigning priors, are considered. To compare the behavior of the popular BYM and BYM2 models in single and joint models, we perform a simulation study with different types and proportions of zero, which are common in medical studies. As an application, the models are applied on data of male breast cancer in Iran. The models can be compared according to the deviance information criterion (DIC) and the logarithmic scoring (LS) suggested by Spiegelhalter et al. [16] and Gneiting and Raftery [17]. 

The main framework of the paper is as follows: Section 2 gives a brief explanation on spatial models and ZIP models with different types of excess zeros, and then the spatial joint model is introduced practically. Section 3 and Section 4 give explanations about simulation study and real data. Section 5 shows the result of different simulation scenarios and mapping male breast cancer in Iran in 2014, and Section 6 and Section 7 are the discussion and conclusions.

## 2. Materials and Methods

Disease mapping is used to identify the spatial pattern of diseases and characteristics of areas and to discover unusual areas with high or low relative risk. In spatial studies, it is assumed that adjacent areas (e.g., administrative areas such as county, state, province) are more similar in environmental characteristics. These characteristics have similar effects on disease prevalence and incidence rates. Therefore, in spatial modeling, information obtained from neighboring areas should be used. For this purpose, Bayesian hierarchical models are proposed. In most published studies, Poisson distribution is used because the numbers of diseases or mortality cases are discrete and rare relative to the target population [1].

### 2.1. The Spatial Models

The number of occurrences or deaths for each area i, i=1,…,n, is denoted by $o_{i}$. The observation $o_{i}$ follows a probability function (likelihood function) as:

(1) $$p\left(o_{i}|\theta_{i}\right)=\frac{\exp\left(-E_{i}\theta_{i}\right){\left(E_{i}\theta_{i}\right)}^{o_{i}}}{o_{i}!}$$

so that $E_{i}$ and $\theta_{i}$ are the expected number (offset) and relative risk. The main goal is modeling $\theta_{i}$, so using canonical link function a linear model is used as:

(2) $$log\left(\theta_{i}\right)=\alpha +x_{i}^{T}\delta +\gamma_{i}+log\left(E_{i}\right)$$

where α, $x_{i}^{T}$, δ and γ denote intercept or average of incidence rate, region covariates, regression parameters and random effects, respectively. Priors on α and δ are specified non-informative, usually normal distribution with a large variability. Priors for random effects will be described in the next subsection.

#### 2.1.1. BYM Model

At first, Besag et al. [18] proposed a combination of spatially structured and unstructured effects in the BYM model as

(3) $$log\left(\theta_{i}\right)=\alpha +x_{i}^{T}\delta +v_{i}+u_{i}+log\left(E_{i}\right),$$

where $v_{i}\sim N\left(0,\tau_{v}^{-1}I\right)$ represents the spatial unstructured random effect and $\tau_{v}^{-1}$ the marginal variance. In contrast, $u_{i}\sim N\left(0,\tau_{u}^{-1}Q^{-}\right)$ is the spatial structured random effect and is modeled under the class of intrinsic Gaussian Markov random field models. Q denotes the precision matrix (neighboring matrix), and $Q^{-}$ is the generalized inverse of Q. The marginal variances are $\tau_{u}^{-1}{\left[Q^{-}\right]}_{ii}$, which are dependent on the neighboring matrix Q. In this model, covariance matrix is $Var(\gamma \;|\tau_{v},\tau_{u})=\tau_{v}^{-1}I+\tau_{u}^{-1}Q^{-}$. Gamma priors with small rate parameters are commonly assigned to $\tau_{v}$ and $\tau_{u}$. Here, Gamma (1, 0.01) is considered.

In the BYM model, components v and u can not be interpreted independently because the independent random effect can be seen in the spatial random effect partially for the case where there is no spatial dependence. To resolve this issue, priors should be heavily dependent [8]. Hence, Dean et al. [19] replaced the precision parameters of the BYM model with a common precision parameter and added a mixing parameter. On the other hand, in the Dean model, priors cannot be transferred from one graph to another because the marginal variance of spatial dependence effect depends on the graph. Then, to remove the effect of graph structure on precision parameter priors, Simpson et al. [8] developed the Dean model by scaling the neighborhood matrix. They also introduced a new method, termed penalized complexity (PC) priors, to assign priors to the hyperparameters. PC priors are obtained from penalizing the complexity caused by deviation of the base model. 

#### 2.1.2. BYM2 Model

Simpson et al. [8] re-parameterized the BYM model with the mixing parameter φ∈[0,1] and the precision parameter $\tau_{\gamma }$. They also scaled the spatial structured effect to facilitate interpretation and transition priors. The BYM2 is as:

(4) $$log\left(\theta_{i}\right)=\alpha +x_{i}^{T}\delta +\frac{1}{\sqrt{\tau_{\gamma }}}\left(\sqrt{1-\varphi }v_{i}+\sqrt{\varphi }u_{i}^{*}\right)+log\left(E_{i}\right).$$

here, $u_{i}^{*}$ is the scaled structured effect. The generalized variance is equal to $1/\tau_{\gamma }$. Thus, $\tau_{\gamma }$ shows the marginal deviation from the model without random effects independent of the graph. The covariance matrix is defined as $Var(\gamma \;|\tau_{\gamma },\;\varphi )=\tau_{\gamma }^{-1}\left(\left(1-\varphi \right)I+\varphi Q_{*}^{-}\right)$ with $Q_{*}^{-}$ the scaled precision matrix. Therefore, using the standardized $Q_{*}^{-}$, the marginal precision from v and $u^{*}$ are $\left(1-\varphi \right)/\tau_{\gamma }$ and $\varphi /\tau_{\gamma }$, respectively and are interpretable independently. In the BYM2 model, PC priors can be applied to φ and $\tau_{\gamma }$. We refer to Simpson et al. [8] and Riebler et al. [20] for more details about BYM2 model and PC priors. 

### 2.2. Joint Model

The focus is on disease mapping of count data with extra zeros (often with 50–80 percent zeros in the data). The Zero-inflated Poisson distribution is used instead of Poisson distribution when there are extra zeros in data. Extra zeros are divided into two types: Structural and non-structural zeros (sample zero). The structural zeros come from reality (the number of occurrences in area is reported zero based on reality). However, sample zeros come from chance (the number of occurrences in area is reported zero based on chance or mistake). In this section, we explain zero-inflated Poisson models type1, type0 and extend spatial modeling to a joint model. 

#### 2.2.1. Zero-Inflated Poisson Model Type1

Here, the number of occurrences or deaths (observations) is assumed to follow a probability function (likelihood function) as

(5) $$p\left(o_{i}|\theta_{i},p_{i}\right)=(1-p_{i})I\left(o_{i}=0\right)+p_{i}\frac{\exp\left(-E_{i}\theta_{i}\right){\left(E_{i}\theta_{i}\right)}^{o_{i}}}{o_{i}!}$$

for each area i, i=1,…,n. This probability function is a mixture of proportion of structural zeros $\left(1-p_{i}\right)$ and sample zeros $\left(p_{i}\right)$. The Poisson distribution is applied to count data with sample zeros. The model includes both types of zero and is known as “Type1” in INLA texts and packages. 

#### 2.2.2. Zero-Inflated Poisson Model Type0

When only structural zeros may occur in data, observations follow a probability function as

(6) $$p\left(o_{i}|\theta_{i},p_{i}\right)=(1-p_{i})I\left(o_{i}=0\right)+p_{i}I\left(o_{i}>0\right)\frac{\exp\left(-E_{i}\theta_{i}\right){\left(E_{i}\theta_{i}\right)}^{o_{i}}}{o_{i}!}.$$

The Poisson distribution is applied for non-zero counts (truncated Poisson), and $\left(1-p_{i}\right)$ is defined as the proportion of structural zeros. The model includes only structural zeros and is known as “Type0” in INLA texts and packages.

The main interest is now in the modeling two latent fields $p_{i}$ and $\theta_{i}$ using canonical link function $logit\left(p_{i}\right)$ and $log\left(\theta_{i}\right)$. As usual the linear predictor for the Poisson is log transformation, and for the Bernoulli it is logistic transformation. It should be noted that $log\left(\theta_{i}\right)$ and $logit\left(p_{i}\right)$ can include a different set of covariates $x_{i}$, $y_{i}$ and random effects [1]. However, in the spatial joint model the spatial random effect (γ) is shared on both $log\left(\theta_{i}\right)$ and $logit\left(p_{i}\right)$. The models for the latent field are specified as the log link function for $\theta_{i}$ as,

(7) $$log\left(\theta_{i}\right)=\alpha_{O}+x_{i}^{T}\delta +\gamma_{i}+log\left(E_{i}\right)$$

and logit function for $p_{i}$ as

(8) $$logit\left(p_{i}\right)=\log\left(\frac{p_{i}}{1-p_{i}}\right)=\alpha_{z}+y_{i}^{T}\omega +\beta \gamma_{i}.$$

In the last two formulas, $\alpha_{O}$ and $\alpha_{z}$ denote average incidence rate by antilog transformation and average probability of events by antilogit transformation. As prior, normal distribution with mean 0 and precision 0.0001 is assigned to intercepts $\alpha_{O}$ and regression parameters δ, and normal distribution with mean −1 and precision 0.2 is assigned to intercepts $\alpha_{z}$ and regression parameters ω. The parameter β is the scaling parameter that can show the similarity of the spatial pattern between the occurrence and number of occurrences. Prior N(1,10) is used for β because, the more probability of occurrence, the greater the number of occurrences.

The joint model has two likelihoods, one for the occurrence and one for the number of occurrences, so a two-column response matrix is defined. The joint model considers a combination of the Bernoulli distribution and the truncated Poisson distribution. The Bernoulli distribution *(*$z_{i}\sim Binomial\left(p_{i},n_{i}=1\right)$*)* is viewed as the distribution for disease occurrence in area i, and the truncated Poisson distribution *(*$o_{i}\sim Poisson\left(E_{i}\theta_{i}\right))$ is considered for the number of occurrences in area i, given that the disease occurred. Since linear predictor for each response is different, the matrix is specified as misaligned. The number of occurrences in location $n_{i}$ can be zero or a positive number, so the disease occurrence $z_{i}$ at location $n_{i}$ is defined as

$$z_{i}=\{\begin{array}{c} 1\;\\ 0\; \end{array}\;\begin{array}{c} if\;an\;event\;occurs\\ otherwise \end{array}$$

and the number of occurrences $o_{i}$ as

$$o_{i}=\{\begin{array}{c} NA\\ occurrence\;number\; \end{array}\;\begin{array}{c} if\;an\;event\;dosen’t\;occur\\ otherwise. \end{array}$$

In the following, we display response matrix structure Y and code of the joint model within the R-INLA package with PC priors for BYM2 model. In this code, phi=φ and $\mathrm{pc}.\mathrm{prec}=1/\sqrt{\tau_{\gamma }}$. See the website http//www.r-inla.org/ that provides documentation and programming commands for INLA package.


$\left[\begin{array}{c} \begin{array}{cc} Z & O \end{array}\\ \begin{array}{cc} z_{1} & NA \end{array}\\ \begin{array}{cc} z_{2} & NA \end{array}\\ \begin{array}{cc} z_{3} & NA \end{array}\\ \begin{array}{cc} \vdots & \vdots \end{array}\\ \begin{array}{cc} z_{n} & NA \end{array}\\ ----\\ \begin{array}{cc} NA & o_{1} \end{array}\\ \begin{array}{cc} NA & o_{2} \end{array}\\ \begin{array}{cc} NA & o_{3} \end{array}\\ \begin{array}{cc} \vdots & \vdots \end{array}\\ \begin{array}{cc} NA & o_{n} \end{array} \end{array}\right]\overset{for\;example\;Y=}{\to }\;\left[\begin{array}{c} \begin{array}{cc} Z & O \end{array}\\ \begin{array}{cc} 1 & NA \end{array}\\ \begin{array}{cc} 0 & NA \end{array}\\ \begin{array}{cc} 1 & NA \end{array}\\ \begin{array}{cc} \vdots & \vdots \end{array}\\ \begin{array}{cc} 1 & NA \end{array}\\ ----\\ \begin{array}{cc} NA & 2 \end{array}\\ \begin{array}{cc} NA & NA \end{array}\\ \begin{array}{cc} NA & 5 \end{array}\\ \begin{array}{cc} \vdots & \vdots \end{array}\\ \begin{array}{cc} NA & 7 \end{array} \end{array}\right]$

> formula <- Y ~ 1 + mu.z + mu.o +
  f(idx1,model = "bym2", graph=g, scale.model = TRUE, constr = TRUE,
  hyper=list(phi = list(prior = "pc",
  param = c(0.5, 2/3), initial = 3),
  prec = list(prior = "pc.prec",
  param = c(1, 0.01),
  initial = 1.5))) +
  f(idx2, copy=’idx1’, fixed=FALSE)
> r.bym2 <- inla(formula, family=c(’binomial’, ’poisson’),
  data= Diseasedata, E=E, verbose=F,
  control.predictor=list(compute=TRUE, link=fam),
  control.compute=list(dic=TRUE, cpo=TRUE))


We implemented the mentioned models under a simulation study and on a real example via the stable version of the package INLA under R3.4.4. For spatial modeling or model process, the BYM and BYM2 models were considered. We can compare results by the DIC and LS based on the conditional predictive ordinate (CPO) as the model selection criteria. A smaller value of the DIC and LS shows that the model is better. Note that when we deal with two likelihoods, DIC and LS are the sum of local deviance information criteria and LSs in joint model [1].

## 3. Simulation Study

We simulated 200 datasets under 60 different scenarios to investigate the behavior of mentioned spatial models on the ZIP models type0, type1 and the joint model. Generally, non-contagious diseases, like cancers or contagious diseases with low prevalence like HIV, have low expected numbers. Therefore, we simulated with low values of E=1 and 5. In contrast, the epidemic wave of some contagious diseases has high prevalence in some areas but may not be observed in other areas like influenza. Therefore, we simulated with higher expected values of E=15, 60 and 200. Data are generated under the same pattern proposed by Riebler et al. [20] with two types of zero (type0 and type1) and proportions of zeros (P=0.50 and P=0.70), five different expected numbers (E=(1, 5, 15, 60, 200)) and three levels of risk; constant risk, spatially unstructured risk and spatially structured risk. Since truncated Poisson is used in joint model, the pattern of data generation for the joint model is similar to the type0 model. 

Consistent with Riebler et al.’s study, we used the neighborhood structure of Sardinia in simulations. Sardinia is a large Italian island in the Mediterranean Sea with 366 regions, suitable for testing methodology. The posterior mean estimates are considered for probability of zero, the two hyperparameters $\left(1/\sqrt{\tau_{\gamma }}\;\mathrm{and}\;\varphi \right)$ and β scaling parameter for the joint model as well as DIC and LS. 

## 4. Case Study: Male Breast Cancer in Iran

Male breast cancer is defined as a lump that can grow out of control in the chest. Breast cancer is almost 1.5% to 2.5% of all cases of malignancies in men [21]. Although this cancer is rare, its incidence rate is increasing [22]. More than 2000 cases of male breast cancer have been reported in the United States in 2012 [23]. Clinical features to breast cancer in males are like those in women [24]. Risk factors, such as positive family history; advanced age; obesity; BRCA2 mutations; conditions that increase estrogen to androgen ratio (such as liver diseases or prostate cancer treated with estrogens) and environmental factors, including high temperature, pollutants, aromatic hydrocarbons, radiation, etc., increase the incidence of male breast cancer [24,25]. 

Iran is a country in Western Asia and covers 1,648,195 km^2^ of land. Cancers are the third highest cause of mortality in this country, so controlling and preventing cancers are important issues in the field of health in this country. According to the report of the Statistical Center of Iran (SCI) in 2015, Iran has almost 78 million inhabitants (51% males) and is subdivided into 429 administrative areas called counties.

The Iranian National Population-based Cancer Registry (INPCR) recorded cancers according to coding system of the International Classification of Diseases for Oncology ICD-O-3. The INPCR covered 98% of the total population in 2014 [26]. The number of reported cases of male breast cancer are based on code C50 and had a low incidence in Iran. Only 303 cases were reported for 429 counties in 2014 with the average of 0.71 per region. Unfortunately, regarding the importance of spatial patterns and environmental risk factors in the diagnosis and prevention of cancers, there is no geographic study on male breast cancer in Iran.

**Figure 1:**
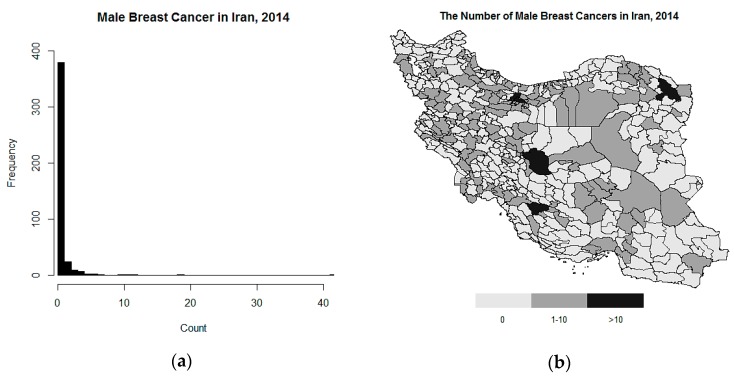
The histogram (**a**) and the map (**b**) of the number of male breast cancer cases in Iran by county level.

We decided to fit the mentioned models on dataset of the male breast cancer to obtain estimates of incidence rate in Iran, in 2014, over the population at risk aged >20 years. The data on male breast cancer were obtained from the INPCR. The frequency of disease was zero in many counties. A histogram of the number of cases and the map are shown in Figure 1. For 299 (69.7%) regions, no cases were reported or discovered, and 126 (29.4%) regions had values between 1–10 cases. Tehran, the capital of Iran, and Mashhad have the highest population and the largest number of cases were reported, 42 and 19 cases, respectively. The mean age was 60.6 ± 15.45 (range 20–93) and male breast cancer was diagnosed for about 2.3% of all cases of breast cancer in 2014. The population at risk is considered based on the 2014 census of the Statistical Center of Iran (SCI). The best model is selected by DIC and LS, because there is no information about whether zeros are true or false.

## 5. Results

### 5.1. The Simulation Study

We performed a simulation study to assess the behavior of the BYM2 model on single models (the zero-inflated data of type0 and type1) and on the joint model. The results of type0, type1 and the joint model are almost similar for both proportions of zeros *P* = 0.50 and *P* = 0.70. Our focus is on the joint model, so we display the result of the joint model as a sample of simulation study results. 

Table 1 shows the posterior mean estimates for intercepts, hyperparameters $\left(1/\sqrt{\tau_{\gamma }}\;\mathrm{and}\;\varphi \right)$, scaling parameter β and average standard deviations (showed in parentheses) of the joint model. The intercepts have non-informative priors; thus, they are estimated closer to the likelihood. We need to apply $\exp(\alpha_{z})/\left(1+\exp(\alpha_{z}\right))$ transformation and $\exp(\alpha_{o})$ transformation on the intercepts $\alpha_{z}$ and $\alpha_{o}$ to obtain the average probability of occurrence and average incidence rate. Here, we expect $\exp(\alpha_{o})$ to be estimated close to 1, and $\exp(\alpha_{z})/\left(1+\exp(\alpha_{z}\right))$ to be estimated close to 0.50 for *P* = 0.50, and 0.30 for *P* = 0.70. Intercepts are well-estimated in each three levels. The parameter β has informative prior, thus it is estimated closer to the prior distribution. All βs confirm the similarity of spatial pattern between occurrence and number of occurrences because the credibility intervals of βs do not contain zero. The results of the estimates for $1/\sqrt{\tau_{\gamma }}$ are almost reasonable for all levels, but there is no change, even by increasing the expected numbers. Datasets at constant level have no information about φ, so mixing parameter $\hat{\varphi }$ is estimated based on the prior distribution. In other levels, mixing parameters could be well-estimated, especially when expected numbers are bigger. The results for *E* = 1 give no clear information at each three levels of risk. 

In the second step, despite the advantages of the BYM2 model compared to the BYM model, we would like to compare DIC and LS average values of the BYM2 and BYM models. We included spatial effect for the number of occurrences in single models. According to Table 2, deviance information criteria of type0 and type1 of the BYM2 in all scenarios are smaller or almost equal to that of the BYM model. In the joint model, deviance information criteria of the BYM2 are slightly lower than deviance information criteria of BYM, in most cases. However, LSs of type0, type1 and the joint model of BYM2 have a small difference compared to the BYM model. 

### 5.2. Analysis of Male Breast Cancer in Iran

We fitted the BYM and BYM2 models on male breast cancer data and reported the DIC and LS values for the joint model (see Table 3). Eighty five percent of areas have the expected number between (0–1) and the average of expected number is 0.70. The output of the joint model shows that $\hat{\varphi }=0.56$, which means 56% tendency to spatial dependence and 44% tendency to over-dispersion or spatial independence. $\hat{\beta }=0.95$ with credibility interval (CI) = (0.08–0.96) shows that spatial pattern of occurrence and the number of occurrences are similar in the joint model. In addition, $1/\sqrt{{\hat{\tau }}_{\gamma }}=0.20$, which shows the marginal deviation from constant level α, independent of the graph. In the joint model, DIC and LS of the BYM2 are slightly smaller than those of the BYM model. In addition, we checked and saw a reduction in DIC and LS in joint model compared to the sum of them in single models. 

On the other hand, we would like to compare DIC and LS values of the BYM2 and BYM models for type1 and type0 models on the real data. As usual, we included spatial effect for the number of occurrences in type0 and type1 models. According to Table 4, in the type0 and type1 models, the DIC and LS of BYM2 model are slightly different from those of BYM model. According to model selection criteria, DIC and LS, type1 model is more appropriate than type0 model for male breast cancer data. The output of models shows that zero probability is estimated 0.08 using type1 and 0.70 using type0. Type1 gives lower probability owing to some zeros covered by the Poisson distribution. 

**Figure 2:**
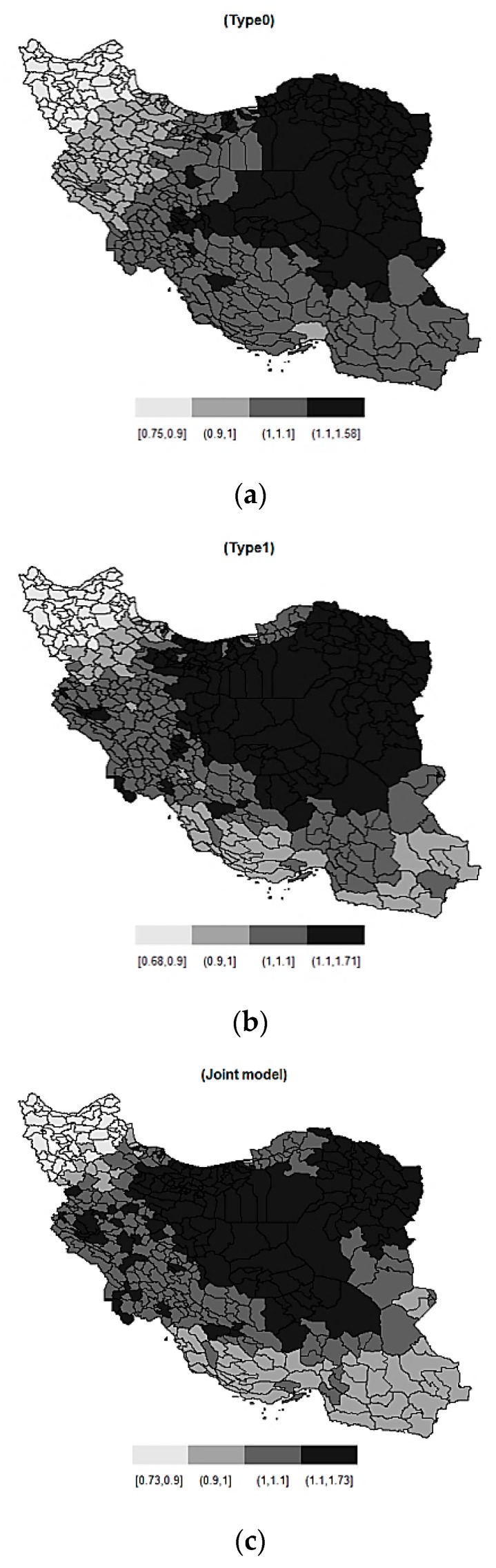
The maps for the posterior relative risk of male breast cancer data for 429 counties using the BYM2 model for type0 (**a**), type1 (**b**) and the joint model (**c**).

According to the results and advantages of the BYM2, we performed the male breast cancer mapping based on the BYM2 model. Figure 2 shows maps of the estimated relative risk (exp(γ)) of male breast cancer across 429 counties in Iran using the BYM2 model for type0, type1 and joint model.

## 6. Discussion

The major aim of this paper was mapping data with extra zeros using the joint model by INLA method. Further, we studied the behavior of the popular BYM model and BYM2 model to analyze count data with different proportions and types of zeros. The BYM2 model has three main advantages: (1) Priors are transferable by scaling the precision matrix—after scaling, a fixed hyperprior for the precision parameter gives the same amount of smoothing if the graph changes; (2) interpretations of the hyperparameters are clear—the precision parameter shows deviation from a constant risk without depending on the graph structure, and the mixing parameter shows variability between the spatial dependence component and the spatial independence component and (3) the model structure gets prepared to assign PC priors—these priors shrink toward simpler models, such as a model with constant risk or a model without spatial correlation, so we focused on scaling and PC priors using the BYM2 model in simulation study and real data.

Riebler et al. [20] investigated on the BYM2 model with details under the simulation study with different scenarios using Poisson distribution and common disease prevalence. Furthermore, they compared the BYM2 model with usual spatial models in disease mapping and proved that the BYM2 model performs well. They used the INLA method for fitting models instead of MCMC. 

Our simulation study on excess zeros showed the BYM2 model’s ability to shrink toward true values in different risk surfaces. This study also showed that the joint model in combination with the BYM2 model performs well, without convergence problems, by INLA method. According to selection criteria, DIC and LS, there was a slight difference between the BYM model and BYM2 model. Therefore, the BYM2 model using INLA is preferred to BYM model for mapping zero-inflated data. The selection between single model and the joint model is dependent on the nature of the data. When data come from two distributions and are influenced by common factors such as location and time, joint models are better. On the other hand, the best model can be chosen by model selection procedures. For preference between single model and joint model, we can fit two single models for each response with spatial component and then obtain the sum of DIS and LS of single models and compare with DIC and LS of joint model. In this study, a small number of datasets in the simulation study were selected at random, and in each of these datasets, we saw a large reduction in DIC and LS for joint model compared to separate models.

Finally, Bayesian spatial joint model, including the BYM and BYM2 models, and type0 and type1 models were applied on male breast cancer data in 429 counties in Iran. Regarding the advantages of the BYM2 model compared to the BYM model, maps were displayed based on the BYM2 model. The maps show that most areas with a high incidence rate of male breast cancer are in the central and eastern parts of Iran. The map of joint model shows that West Azerbaijan province (located in the northwest of Iran) and Khorasan Razavi province (located in the east of Iran) had the lowest (0.73) and highest (1.73) posterior means of the relative risk in 2014, respectively. 

According to the Natural Center for Climatology of Iran, northwest provinces of Iran are mountainous and have cool weather. However, most areas in east and central Iran have warm and dry weather, and people are exposed to direct sunlight. Many people in these counties have outdoor occupations (such as working on farms), have special diets (such as eating spicy food) and consume unique opiate (Naswar). Unfortunately, there is no study with a focus on spatial modeling that identifies the geographic pattern of male breast cancer in Iran. Hence, this paper was designed to fill this gap. However, due to the lack of an appropriate registration method, we performed a simple model without covariates and environmental factors. We hope that in the near future, the quality of cancer data registration improves, making it possible to investigate environmental causes by statistical analysis.

Further researches, in order to expand models to other fields, can investigate the BYM2 behavior in spatiotemporal models to zero-inflated data by considering other types of zeros and/or other distributions instead of the Poisson distribution.

## 7. Conclusions

This study showed that the joint model for mapping of zero-inflated data in combination with the spatial models performs well, without convergence problem, by INLA method. According to selection criteria there are slightly difference between the BYM and BYM2 models. Therefore, the spatial joint model in combination with the BYM2 model using INLA can be used for disease mapping of zero-inflated data.

## Figures and Tables

**Table 1:** The posterior mean estimates and average standard deviations (in parentheses) of the joint model based on 200 simulations at each three levels of risk.

*P*	*E*	$\alpha_{z},\alpha_{o}$	$1/\sqrt{{\hat{\tau }}_{\gamma }}$	$\hat{\varphi }$	β
**Constant risk $\left(1/\sqrt{\tau_{\gamma }}=0\;\&\;\varphi =0\right)$**					
0.50	1	−0.02(0.10), −0.06(0.12)	0.17(0.14)	0.39(0.27)	1.12(0.29)
	5	0.16(0.10), −0.03(0.03)	0.07(0.04)	0.36(0.29)	0.98(0.30)
	15	0.16(0.10), 0.01(0.02)	0.02(0.008)	0.25(0.23)	0.93(0.32)
	60	0.12(0.10), −0.01(0.01)	0.01(0.004)	0.26(0.24)	0.91(0.33)
	200	−0.07(0.10), 0.004(0.006)	0.02(0.02)	0.36(0.23)	0.85(0.36)
0.70	1	−0.91(0.11), −0.21(0.17)	0.05(0.02)	0.28(0.23)	0.99(0.31)
	5	−0.53(0.11), 0.001(0.04)	0.03(0.01)	0.26(0.24)	0.95(0.32)
	15	−0.82(0.11), −0.002(0.03)	0.07(0.05)	0.21(0.22)	0.96(0.32)
	60	−0.85(0.11), −0.02(0.01)	0.02(0.01)	0.27(0.26)	0.95(0.32)
	200	−1.03(0.12), −0.01(0.008)	0.01(0.01)	0.31(0.28)	0.92(0.32)
**Spatially unstructured risk $\left(1/\sqrt{\tau_{\gamma }}=0.5\;\&\;\varphi =0\right)$**					
0.50	1	0.12(0.11), 0.08(0.11)	0.39(0.04)	0.32(0.25)	1.01(0.30)
	5	−0.11(0.11), −0.20(0.07)	0.40(0.03)	0.07(0.08)	1.11(0.26)
	15	0.16(0.11), −0.18(0.05)	0.49(0.0.4)	0.06(0.07)	1.12(0.21)
	60	−0.04(0.11), −0.12(0.05)	0.45(0.04)	0.05(0.05)	1.13(0.14)
	200	0.07(0.11), −0.13(0.04)	0.43(0.05)	0.03(0.03)	1.32(0.08)
0.70	1	−1.01(0.12), −0.43(0.26)	0.50(0.03)	0.14(0.13)	1.28(0.26)
	5	−0.82(0.12), −0.20(0.10)	0.51(0.04)	0.06(0.08)	1.20(0.25)
	15	−0.83(0.12), −0.15(0.07)	0.48(0.03)	0.08(0.14)	1.06(0.22)
	60	−0.70(0.11), 0.02(0.05)	0.48(0.02)	0.08(0.02)	1.18(0.10)
	200	−0.98(0.12), −0.16(0.06)	0.42(0.04)	0.06(0.07)	1.17(0.18)
**Spatially structured risk $\left(1/\sqrt{\tau_{\gamma }}=0.5\;\&\;\varphi =1\right)$**					
0.50	1	0.08(0.11), −0.08(0.14)	0.34(0.04)	0.18(0.17)	1.11(0.27)
	5	0.19(0.11), −0.06(0.05)	0.52(0.03)	0.89(0.08)	1.60(0.21)
	15	0.02(0.11), −0.10(0.03)	0.55(0.03)	0.84(0.11)	1.46(0.22)
	60	−0.02(0.11), −0.06(0.02)	0.53(0.03)	0.89(0.08)	1.37(0.21)
	200	−0.01(0.11), −0.07(0.02)	0.48(0.04)	0.95(0.05)	1.48(0.21)
0.70	1	−0.92(0.12), −0.28(0.20)	0.36(0.02)	0.26(0.25)	1.04(0.31)
	5	−0.78(0.11), −0.08(0.07)	0.57(0.04)	0.66(0.22)	1.16(0.26)
	15	−0.85(0.11), −0.04(0.05)	0.53(0.04)	0.62(0.20)	1.44(0.23)
	60	−0.95(0.12), −0.09(0.04)	0.51(0.04)	0.87(0.09)	1.43(0.22)
	200	−0.82(0.11), −0.06(0.04)	0.56(0.03)	0.87(0.10)	1.37(0.21)

**Table 2:** Deviance information criterion (DIC) and logarithmic scoring (LS) average values of the Besag, York and Mollié (BYM2 and BYM) models of type0, type1 and the joint model based on 200 simulations at three levels of risk.

*P*	*E*	Type0		Type1		Joint Model	
		BYM2 (DIC,LS)	BYM (DIC,LS)	BYM2 (DIC,LS)	BYM (DIC,LS)	BYM2 (DIC,LS)	BYM (DIC,LS)
**Constant risk $\left(1/\sqrt{\tau_{\gamma }}=0\;\&\;\varphi =0\right)$**							
0.50	1	(881.9,1.20)	(881.5,3.31)	(714.3,0.95)	(714.7,0.95)	(888.0,1.39)	(892.5,1.81)
	5	(1457.0,1.79)	(1459.3,1.79)	(1383.7,1.79)	(1382.3,1.79)	(1281.7,1.19)	(1367.4,1.19)
	15	(1513.4,2.08)	(1517.8,2.08)	(1513.4,2.09)	(1520.5,2.09)	(1595.1,1.39)	(1626.2,1.39)
	60	(1810.4,2.42)	(1818.2,2.43)	(1784.7,2.43)	(1801.4,2.44)	(1706.6,1.62)	(1823.4,1.62)
	200	(2035.4,2.73)	(2054.6,2.75)	(1998.5,2.72)	(2030.6,2.74)	(2062.9,1.82)	(1974.3,1.82)
0.70	1	(683.5,0.93)	(683.2,2.20)	(476.6,0.68)	(476.6,0.68)	(646.2,1.69)	(650.5,1.72)
	5	(933.4,1.27)	(933.3,1.27)	(880.2,1.26)	(883.3,1.26)	(997.8,0.98)	(1074.9,0.98)
	15	(1026.7,1.43)	(1029.3,1.44)	(1095.7,1.44)	(1097.2,1.45)	(1017.0,1.11)	(1092.3,1.11)
	60	(1036.3,1.66)	(1046.0,1.66)	(1228.0,1.65)	(1237.4,1.66)	(1140.7,1.28)	(1206.3,1.27)
	200	(1379.9,1.85)	(1399.0,1.86)	(1407.3,1.83)	(1427.4,1.85)	(1436.6,1.41)	(1205.8,1.41)
**Spatially unstructured risk $\left(1/\sqrt{\tau_{\gamma }}=0.5\;\&\;\varphi =0\right)$**							
0.50	1	(904.5,1.34)	(904.2,2.21)	(709.6,1.01)	(709.8,1.01)	(942.2,1.52)	(949.6,1.92)
	5	(1398.7,2.06)	(1398.6,2.06)	(1360.0,2.01)	(1360.1,2.09)	(1317.4,1.37)	(1454.4,1.36)
	15	(1700.5,2.65)	(1700.2,2.66)	(1757.8,2.67)	(1757.7,2.67)	(1733.4,1.76)	(1784.3,1.76)
	60	(1930.1,3.18)	(1930.1,3.18)	(1984.0,3.19)	(1984.2,3.19)	(1890.0,2.10)	(1921.2,2.10)
	200	(2124.2,3.45)	(2124.2,3.45)	(2177.2,3.44)	(2177.4,3.44)	(2165.2,2.30)	(2222.3,2.30)
0.70	1	(713.2,1.02)	(718.6,1.26)	(570.2,0.73)	(570.4,0.73)	(701.2,2.21)	(657.6,2.83)
	5	(1025.7,1.45)	(1025.6,1.45)	(995.1,1.40)	(995.2,1.56)	(1032.5,1.11)	(1031.7,1.11)
	15	(1120.1,1.80)	(1119.9,1.80)	(1210.5,1.81)	(1210.3,1.81)	(1193.2,1.37)	(1186.5,1.36)
	60	(1387.4,2.10)	(1387.4,2.10)	(1265.5,2.11)	(1265.5,2.11)	(1120.1,1.60)	(1462.8,1.60)
	200	(1492.4,2.28)	(1492.4,2.28)	(1326.3,2.27)	(1326.3,2.27)	(1362.4,1.74)	(1375.1,1.74)
**Spatially structured risk $\left(1/\sqrt{\tau_{\gamma }}=0.5\;\&\;\varphi =1\right)$**							
0.50	1	(860.8,1.43)	(861.4,1.38)	(707.2,1.00)	(707.2,1.00)	(922.6,1.49)	(931.0,1.90)
	5	(1352.5,1.93)	(1351.8,1.93)	(1327.7,1.89)	(1326.3,1.89)	(1363.6,1.29)	(1438.7,1.30)
	15	(1592.9,2.41)	(1592.2,2.41)	(1636.7,2.35)	(1635.8,2.35)	(1642.0,1.62)	(1652.5,1.63)
	60	(1800.8,2.91)	(1800.4,2.91)	(1829.6,2.93)	(1829.3,2.93)	(1772.4,2.01)	(1920.0,2.01)
	200	(2304.5,3.34)	(2304.5,3.34)	(2097.2,3.35)	(2096.7,3.35)	(2223.6,2.29)	(2166.3,2.28)
0.70	1	(698.8,1.02)	(699.1,3.11)	(521.7,0.72)	(522.8,1.26)	(691.1,2.11)	(648.3,2.79)
	5	(941.6,1.36)	(939.9,1.36)	(983.7,1.34)	(983.4,1.34)	(949.2,1.05)	(1019.8,1.05)
	15	(1244.3,1.65)	(1243.7,1.65)	(1119.9,1.61)	(1119.0,1.61)	(1137.5,1.27)	(1158.2,1.28)
	60	(1265.8,1.95)	(1265.4,1.94)	(1289.2,1.95)	(1289.1,1.95)	(1341.9,1.55)	(1252.4,1.57)
	200	(1380.9,2.24)	(1380.8,2.24)	(1499.0,2.21)	(1498.9,2.21)	(1319.2,1.74)	(1500.8,1.74)

**Table 3:** The posterior estimate mean for parameters, DIC and LS values of BYM and BYM2 models of the joint model based on male breast cancer data in Iran, 2014.

	BYM Model	BYM2 Model	BYM Model	BYM2 Model
	$\left(\frac{1}{\sqrt{{\hat{\tau }}_{v}}},\frac{1}{\sqrt{{\hat{\tau }}_{u}}},\hat{\beta }\right)$	$\left(\frac{1}{\sqrt{{\hat{\tau }}_{\gamma }}},\hat{\varphi },\hat{\beta }\right)$	(DIC,LS)	(DIC,LS)
Joint model	(0.09, 0.19, 1.03)	(0.20, 0.56, 0.95)	(797.6, 0.73)	(794.9, 0.72)

**Table 4:** The posterior estimate mean for parameters, DIC and LS values of BYM and BYM2 models of type0 and type1 models based on male breast cancer data in Iran, 2014.

	BYM Model	BYM2 Model	BYM Model	BYM2 Model
	$\left(\hat{p},\frac{1}{\sqrt{{\hat{\tau }}_{v}}},\frac{1}{\sqrt{{\hat{\tau }}_{u}}}\right)$	$\left(\hat{p},\frac{1}{\sqrt{{\hat{\tau }}_{\gamma }}},\hat{\varphi }\right)$	(DIC,LS)	(DIC,LS)
Type0 model	(0.70, 0.11, 0.63)	(0.70, 0.15, 0.51)	(795.5, 0.93)	(796.6, 0.93)
Type1 model	(0.08, 0.09, 0.21)	(0.08, 0.26, 0.61)	(666.9, 0.78)	(668.1, 0.78)
